# Assessing the impact of climate change on water requirement and yield of sugarcane over different agro-climatic zones of Tamil Nadu

**DOI:** 10.1038/s41598-024-58771-8

**Published:** 2024-04-08

**Authors:** V. Guhan, K. Annadurai, S. Easwaran, M. Marimuthu, D. Balu, S. Vigneswaran, C. Navinkumar

**Affiliations:** 1 Meteorological Centre, Hyderabad, India; 2 Sugarcane Research Station, Sirugamani, Tiruchirappalli, Tamil Nadu India; 3 ICAR-KVK, Sirugamani, Tiruchirappalli, Tamil Nadu India; 4 Department of Agricultural Extension, Annamalai University, Chidambaram, Tamil Nadu India; 5 Institute of Forest Genetics and Tree Breeding, Coimbatore, Tamil Nadu India; 6 Department of Agronomy, Adhiparasakthi Agricultural College, Ranipet, Tamil Nadu India

**Keywords:** CANEGRO DSSAT (version 4.7) model, Calibration and validation, Sugarcane crop, Agro Climatic Zones, Elevated temperature, Atmospheric science, Climate change

## Abstract

The DSSAT CANEGRO model was calibrated and verified using field experimental data from five Tamil Nadu Agroclimatic Zones (1981–2022). The genetic coefficients of the sugarcane cultivar (CO-86032) were calculated. R^2^ obtained between measured and simulated stalk fresh mass was 0.9 with the nRMSE (0.01) and RMSE (1.6) and R^2^ between measured and simulated sucrose mass was 0.9 with the nRMSE (0.16) and RMSE (1.2). For yield R^2^ obtained between measured and simulated was 0.9 with the nRMSE (0.01) and RMSE (1.6). As a result, the CANEGRO model may be used to mimic the phenology and yield features of the sugarcane cultivar in Tamil Nadu's Agro Climatic Zones. Temperature increases in Agro Climatic Zones resulted in varying yield reductions, with 2 °C increases causing a 3% loss, 3 °C increases 5%, and 4 °C increases 9%. The Water Requirement rose throughout all of the ACZ due to the high temperature, but to differing degrees. A 2 °C increase often results in an average 4% increase in the WR. 3 °C rise in temperature increased WR to 9% and WR rose by 13% when the temperature was raised by 4 °C.

## Introduction

Sugarcane is a major commercial crop in India. Because the crop goes through several seasons and environmental conditions over its life cycle, all of these elements have a direct influence on the crop's production and maturity. Sugarcane's low productivity is mostly due to late planting in April and May^1,2^. As a result, it is vital to investigate how crop management methods (such as irrigation, fertilizer, and so on) and climatic variation impact yields^1^. Since sugarcane is a commodity with a lengthy growing season, weather variations, such as high summer temperatures and extremely low winter lows, have a significant impact on the crop's final production^3^. Owing to these variations throughout the crop life cycle, forecasting how the crop will react to various stimuli may enable better planning^4–6^ studies aim to increase the efficiency of management and strategic decisions made throughout the cropping season^7^, characterize management alternatives, and create more realistic scenarios for decision analysis simulations and optimizations. Crop yields in crop models are calculated using agronomic management (sowing date, plant population, amount and timing of irrigation and fertilizer applications), weather (radiation, maximum temperature, minimum temperature, rainfall, etc.), soil factors (available water, physical properties, and depth), crop physiological properties (variety and genotype constant), and other factors that hinder crop growth such as pests and diseases. Crop growth models provide a unique opportunity to supplement field trial data since they account for the effect of various variables on yield. There has been relatively little published research on crop growth models for India's sugarcane crop. Using the CANEGRO model^8^, investigated sugarcane yields in response to light interaction inside the green canopy. In India, a variety of research and farmer-level field experiments have been conducted, described in published works, and are available for model testing. As a result, the current study intends to evaluate the DSSAT-CANGRO model version 4.7 in the context of Agro Climatic Zones, especially for the whole Tamil Nadu area.

## Materials and methods

### Location

**Figure 1:**
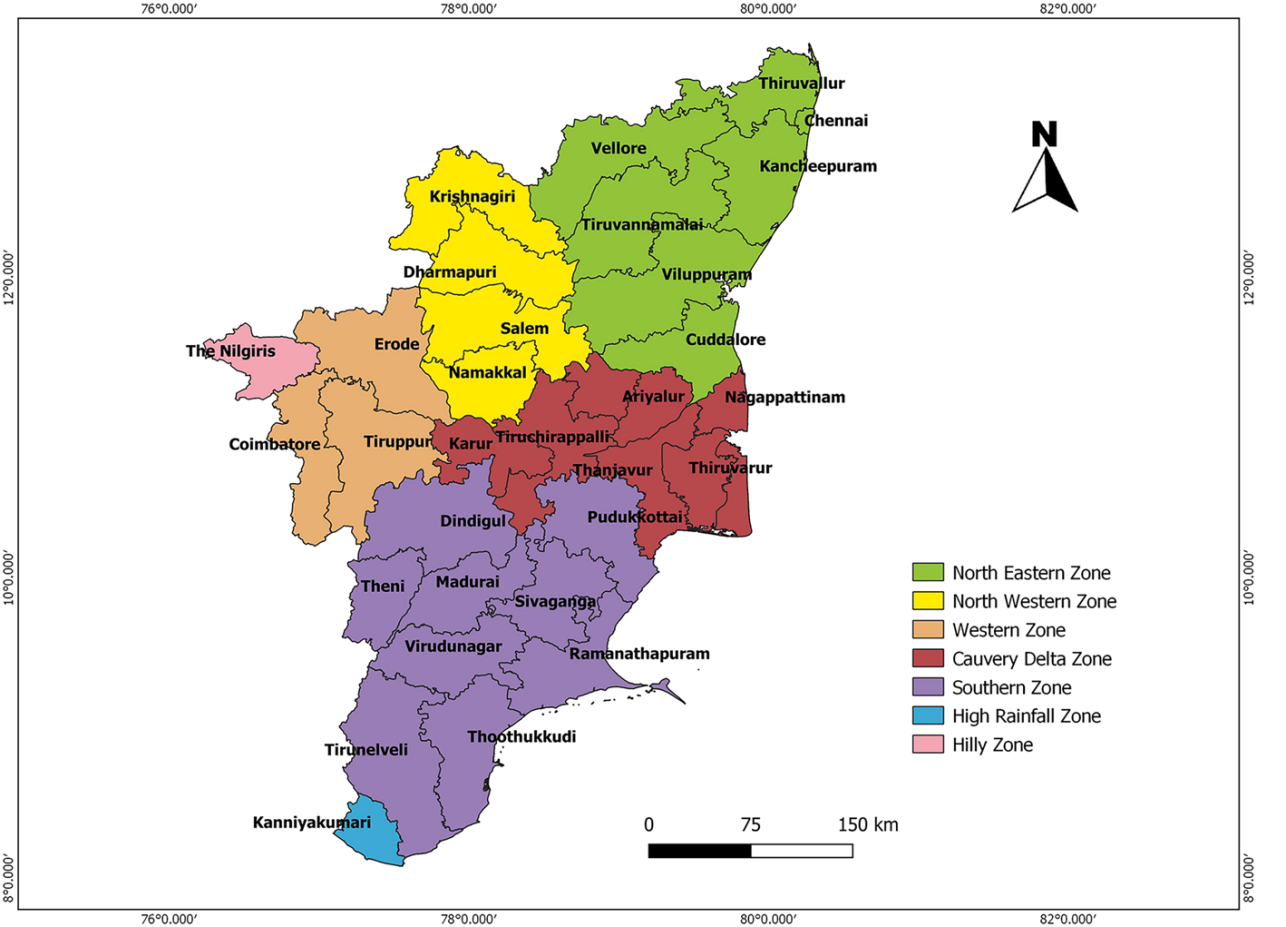
Agro climatic zones of Tamil Nadu.

The diverse terrain of Tamil Nadu, which includes plateaus, hilly areas, and coastal plains, contributes to the state's rich agro-climatic variety. The state is separated into many Agro-Climatic Zones (ACZ) (Fig. 1), with distinct environmental features impacting farming methods in each and the Map were generated using the QGIS Software Version 3.4. The Western (WZ), Northwestern (NWZ), Northeastern (NEZ), Cauvery delta (CDZ), and Southern (SZ) zones are among them. These specific zones were chosen for the study due to their relevance in sugarcane production and their ability to depict the wide range of agroclimatic conditions observed in Tamil Nadu. The study's purpose was to provide a comprehensive knowledge of how varied climatic conditions impact sugarcane productivity and water requirements in these distinct agro-climatic zones.

### Soil data

The soils of the Tamil Nadu sector of the basin were characterized using a 1:50,000 resolution computerized soil map of the state obtained from the Department of Remote Sensing and Geographical Information System, Tamil Nadu Agricultural University (TNAU).

### The influence of climate change on water requirements, WUE, and sugarcane yield in different agro-climatic zones of Tamil Nadu

**Figure 2:**
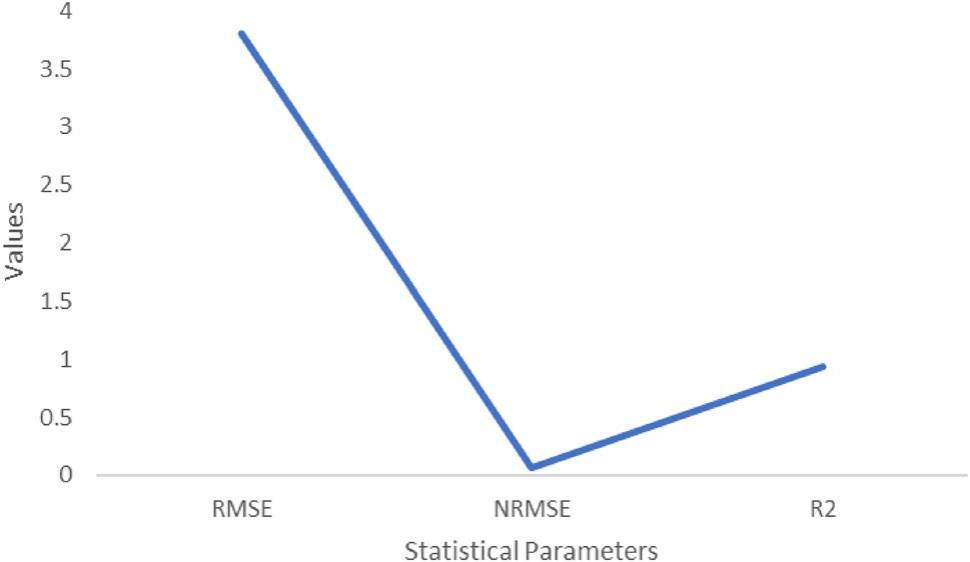
Statistical analysis values for Stalk fresh mass (t ha^−1^).

According to the Season and Crop Report from the Government of Tamil Nadu's Department of Economics and Statistics (2016), cropping districts with high efficiency were drawn across several agro-climatic zones (ACZ) (Fig. 2) in Tamil Nadu, with priority given to places with the most acreage under cultivation for sugarcane. This model has been used to simulate crop water requirements as well as sugarcane yields over the past 43 years. The simulations, which were ran with temperature changes of 2 °C, 3 °C, and 4 °C, enabled a full investigation of the impacts of high temperatures on sugarcane crops.

### Validation and calibration of DSSAT model and sugarcane

Crop simulation models for 42 crops, including sugarcane, are included in DSSAT version 4.7^10^. It was founded by the International Benchmark Sites Network for Agro-technology Transfer (IBSNAT)^9^, as Ref.^1^ also indicate. Utilizing genetic data defined in species, ecotype, and cultivar files, the Canegro model in DSSAT. The crop growth model simulates sugarcane growth during the growing season by combining daily meteorological data with a set of crop, soil, and management characteristics. For this study, daily weather data covering maximum and minimum temperatures, sunshine hours, and rainfall at select stations across Tamil Nadu's Agro Climatic Zones were gathered over a 43-year period (1981–2022).

Soil physical parameters such as albedo, field capacity, wilting point, organic content, bulk density, N-content, and essential soil water in various strata were collected for the research region. The minimum amount of available data sets, such as planting, flowering, and maturity dates; cane yield and sucrose percentage; biomass; cane number (m^−2^); and nitrogen uptake by plants (kg ha^−1^ and %), were also gathered in order to determine the genotype coefficient for various varieties. The different experimental data, multi-year and location data of cane yield, sucrose yield, and maturity dates were also collected for the model’s evaluation. The model requires a large number of genetic coefficients to simulate a sugarcane crop cultivar or variety. There are over 20 genetic coefficients parameters/crop coefficients in the cultivar module, some of which are used to simulate crop phenology, leaf phenology, tiller phenology, growth/biomass partitioning, and sucrose accumulation. The genetic coefficients for the CO-86032 sugarcane cultivar was calculated using the CANEGRO Sugarcane model (DSSAT version 4.7) by repeated interaction in the model computations until a close match between simulated and observed phenology, growth, and yield was established (Table 1). All relevant data for establishing genetic coefficients for the aforementioned sugarcane cultivars were acquired from field tests done by farmers in the Musiri Block of Tiruchirappalli District.

**Table 1:** Genetic coefficients of sugarcane cultivars used in the CANEGRO (DSSAT version 4.7) model.

Genetic coefficient	CO-86032
PARCEmax	8.4
APFMX	0.81
STKPFMAX	0.54
SUCA	0.61
TBFT	27.3
Tthalfo	217
TBase	14
LFMAX	9
MXLFAREA	343
MXLFARNO	12
PI1	88
PI2	165
PSWITCH	12
TTPLNTEM	332
TTRATNEM	200
CHUPIBASE	745
TT_POPGROWTH	665
MAX_POP	24
POPTT16	8
LG_AMBASE	215
DELTTMAX	0.07

## Results and discussion

### Validation and calibration

**Figure 3:**
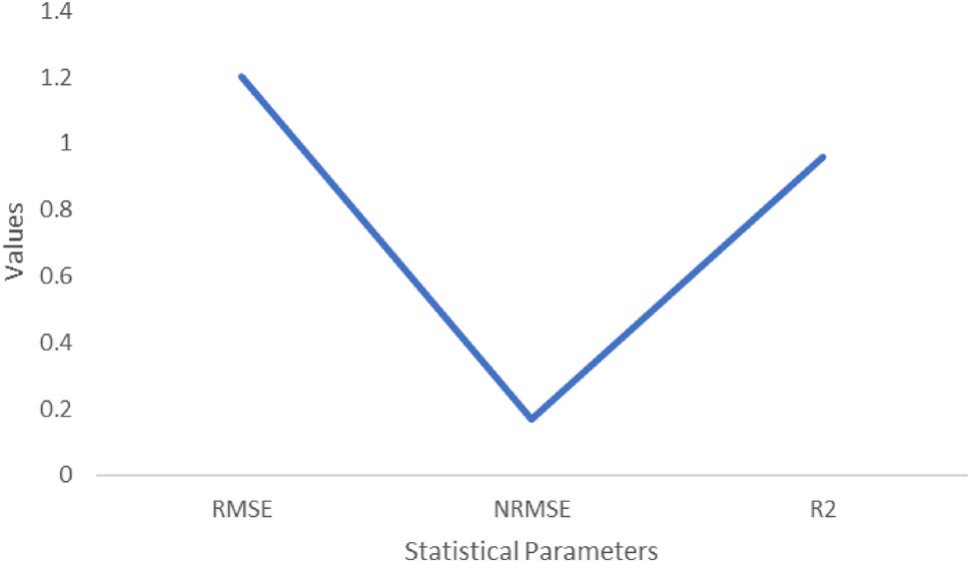
Statistical analysis values for Sucrose mass (t ha^−1^).

**Figure 4:**
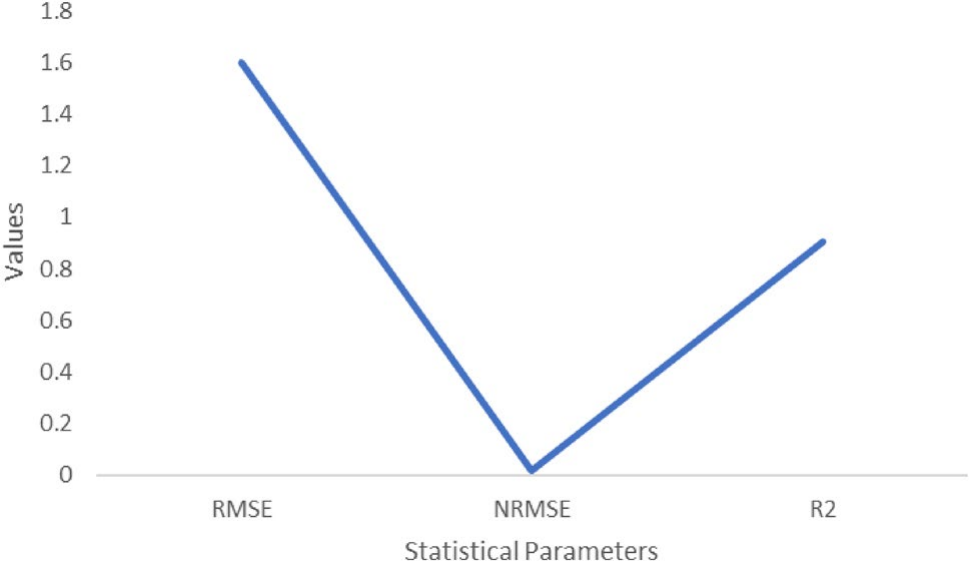
Statistical analysis values for Yield (t ha^−1^).

According to the model statistics for stalk fresh mass, Sucrose mass and yield (Figs. 2, 3, 4) showed a high agreement between observed and model-simulated data (Table 2). For stalk fresh mass the RMSE value is 1.6, NRMSE is 0.016 and R^2^ is 0.9. In case of Sucrose mass, the RMSE is 1.2, NRMSE is 0.16 and R^2^ is 0.9. For Sugarcane yield the RMSE value is 1.6, NRMSE value is 0.;016 and R^2^ is 0.9 respectively. This might be the reason for the decrease in sucrose mass. When temperatures rise, increased photorespiration and the conversion of sucrose into fructose and glucose may result in a reduction in sugar accumulation^11^. According to Ref.^12^, CO_2_ fertilization (520 ppm) resulted in just a midcentury (RCP 4.5) increase in sugarcane productivity in Tamil Nadu.

**Table 2:** Comparison of observed and simulated values.

Parameters	Observed values	Simulated values
Stalk fresh mass (t ha^−1^)	57.4	61.2
Sucrose mass (t ha^−1^)	7.1	8.3
Yield (t ha^−1^)	98.7	100.3

### The effect of varying climate on average sugarcane yield in various agro-climatic zones of Tamil Nadu

**Figure 5:**
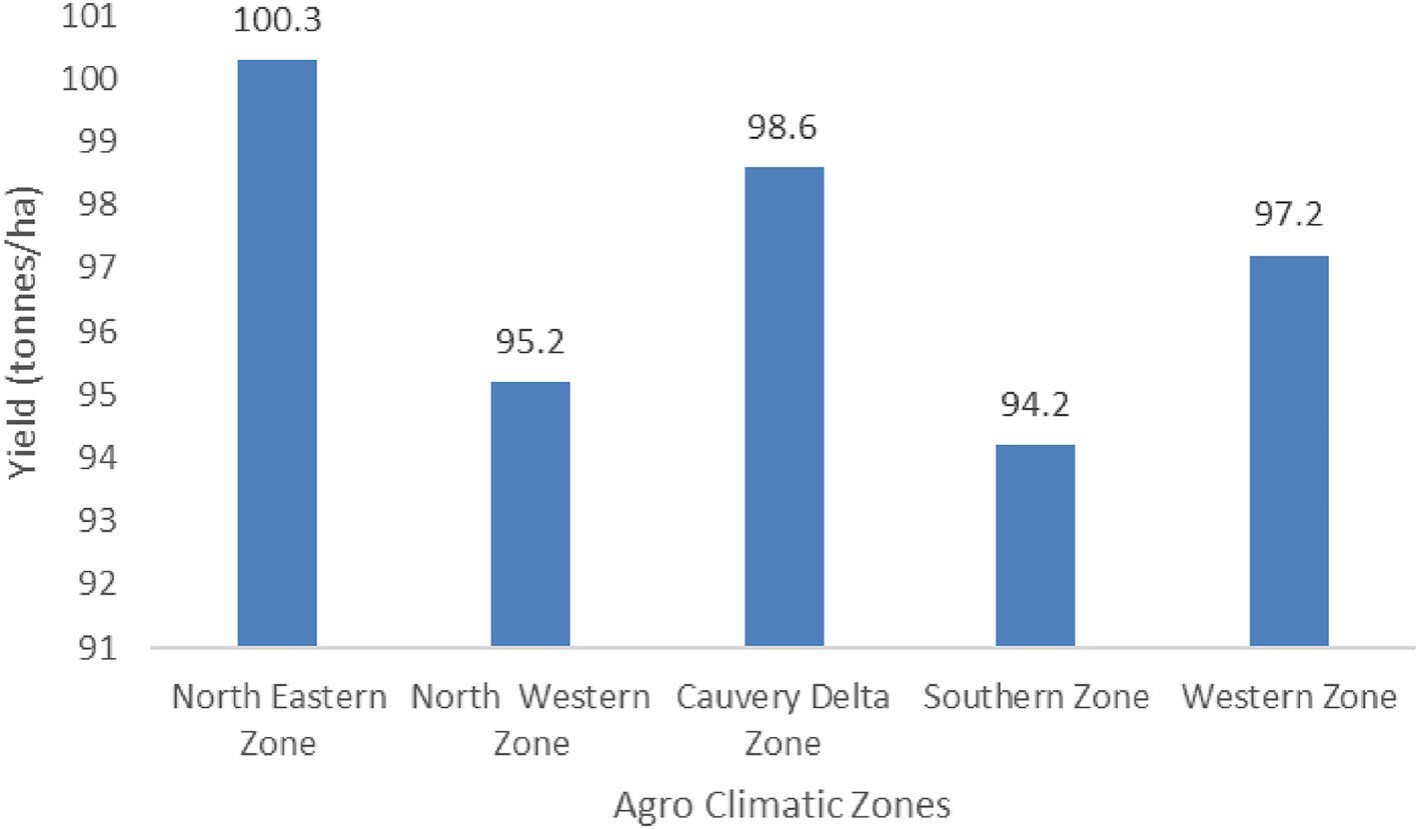
Influence of climate variability on Sugarcane yield (tones/ha) at different Agro climatic zones over Tamil Nadu.

Sugarcane production showed spatial variations among the different agro-climatic zones and inter-annual variability due to the varied climatic conditions (Fig. 5). North Eastern zone showed high yielding potential (100.3 tonnes/ha) with wider annual variability. The Cauvery delta zone stood next in sugarcane productivity (98.6 tonnes/ha). Sugarcane yield was about 97.2 tones/ha in western zone and 95.2 tones/ha in north western zone. Relatively low yield was noticed in the southern zone (94.2 tones/ha). Better crop management techniques could increase sugarcane yield in the Cauvery Delta and North-Eastern Zones by up to 20 and 23%, respectively, according to technical efficiency. Our research also revealed that removing impediments such as a shortage of laborers, high salaries, inadequate water sources, high fertilizer costs, and low cane prices will likely boost the technical efficiency of sugarcane fields^13^.

### Water use efficiency under varied climates in different agroclimatic zones of Tamil Nadu

**Figure 6:**
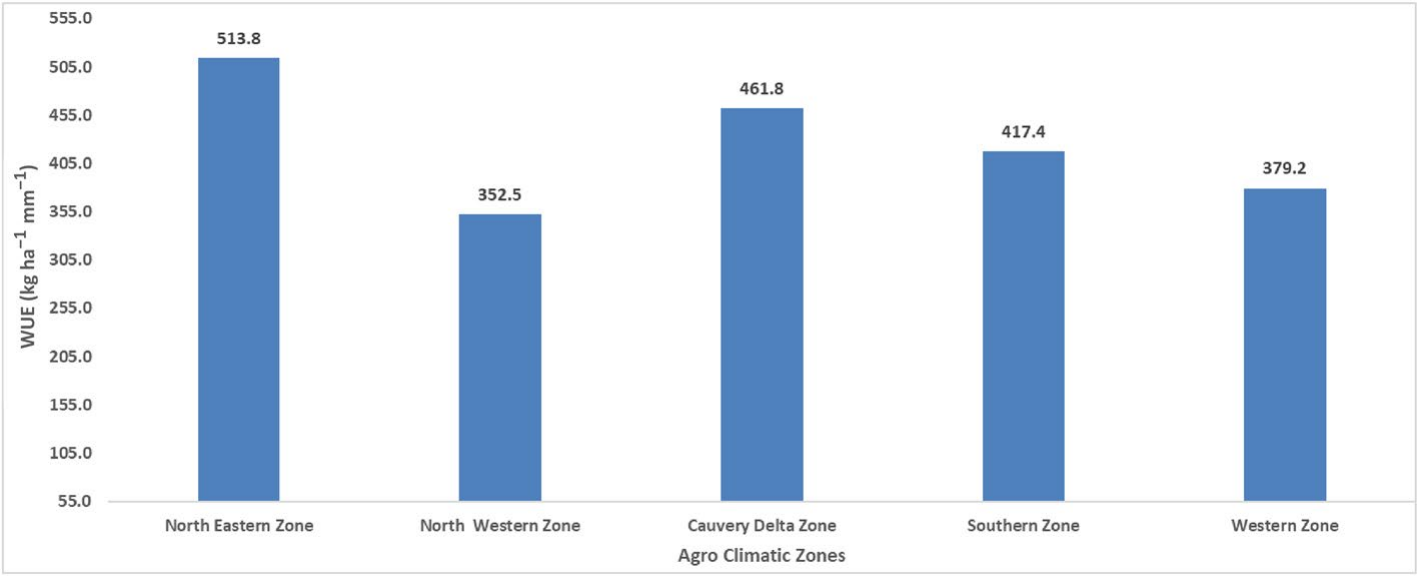
The effect of climate variability on sugarcane WUE (kg ha^−1^ mm^−1^) across agro-climatic zones of Tamil Nadu.

The agro-climatic region's water use efficiency (WUE) was analyzed for the base period from 1980 to 2022. At the agro-climatic zone scale, WUE varied between 352.5 to 513.8 mm. The WR was relatively higher in the Northeastern zone (NWZ) than in other zones (Fig. 6). The type of crop planted locally, as well as the surrounding meteorological circumstances, may affect differences in water usage and production. The variation between the agricultural water requirement and the effective rainfall is represented by the irrigation water requirement^14^.

### Water requirements under varied climates in different agroclimatic zones of Tamil Nadu

**Figure 7:**
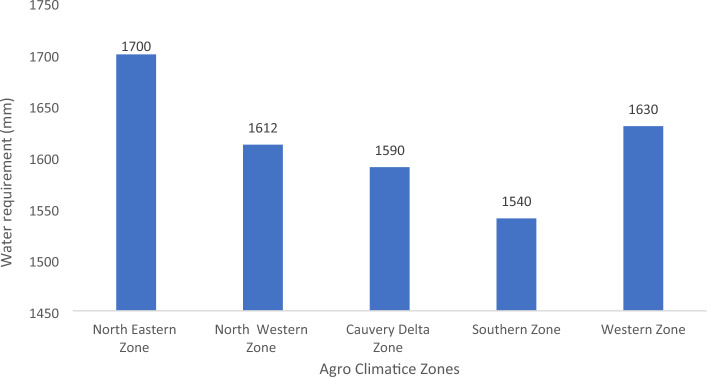
Water requirements under varied climates in different agroclimatic zones of Tamil Nadu.

The agro-climatic region's water requirement (WR) was analyzed for the base period from 1980 to 2022. At the agro-climatic zone scale, WR varied between 1540 to 1700 mm. The WR was relatively higher in the Northeastern zone (NWZ) than in other zones (Fig. 7).

### Elevated temperature affects sugarcane yield and water requirement of sugarcane in the different agro-climatic zone of Tamil Nadu

**Figure 8:**
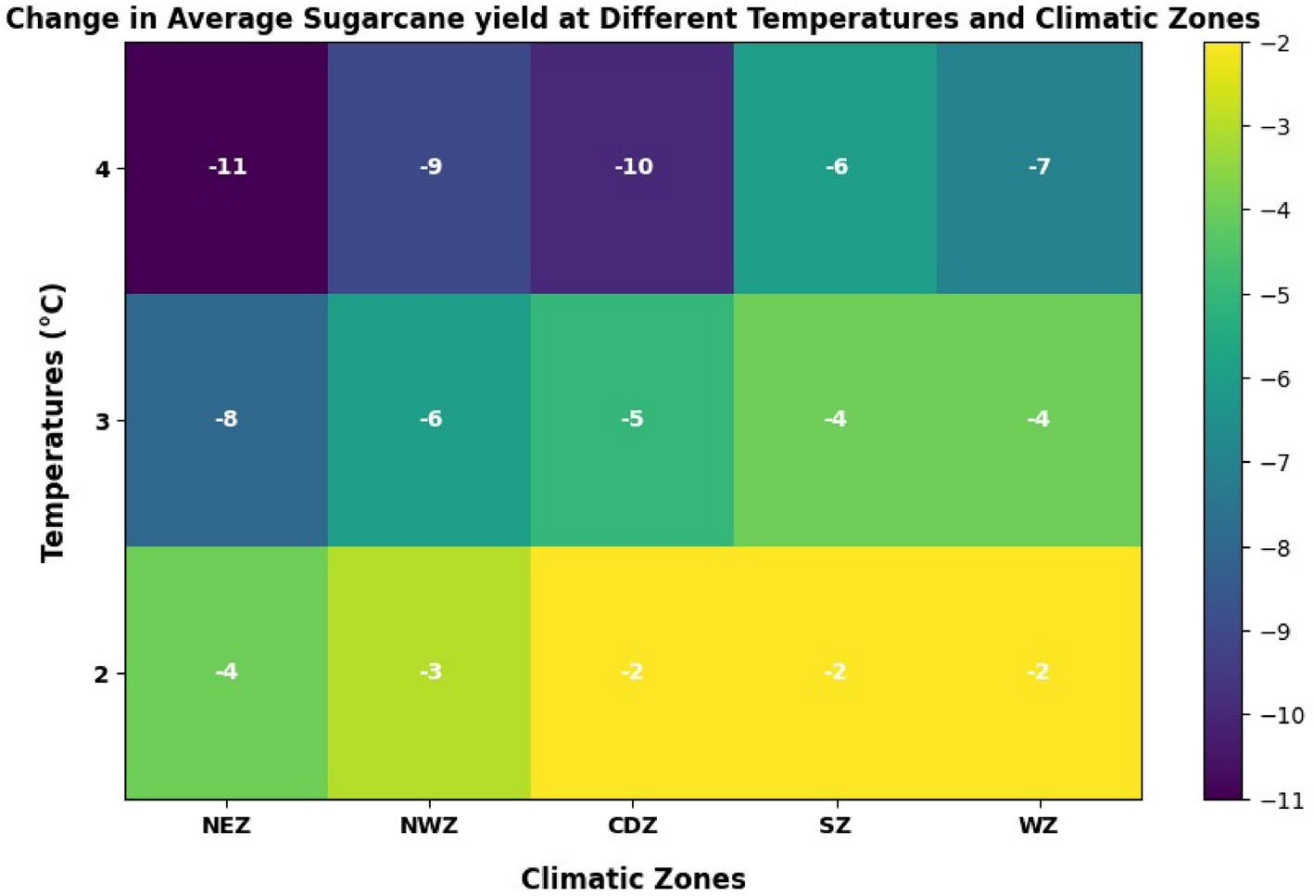
Elevated temperature effects on sugarcane yield.

The results show that sugarcane is more temperature sensitive (Fig. 8). Sugarcane yield reductions owing to higher temperatures are expected to be greater in the NEZ and CDZ. The elevated temperature decreased the yield in all the ACZ at varying magnitudes. An increase of 2 °C tends to reduce the yield by an average of 3%. The elevated temperature by 3 °C resulted in a 5% reduced the yield and the 4 °C temperature rise reduced the yield by 9% (Fig. 8). Reference^15^ hypothesized that increased temperatures and CO_2_ levels will have an impact on plant development and water balance. The requirement for sugar and energy derived from sugarcane is predicted to rise in the future. It is noteworthy that temperature, rainfall (RF), atmospheric CO_2_ concentration, and extreme weather events caused by climate change all have an impact on sugarcane output^10,16,17^. High temperatures and water stress are widely known to have a deleterious impact on crop growth phases (germination, flowering, and maturity)^18^. Weather changes can have an influence on sugarcane yield and quality, especially during critical seasons.

**Figure 9:**
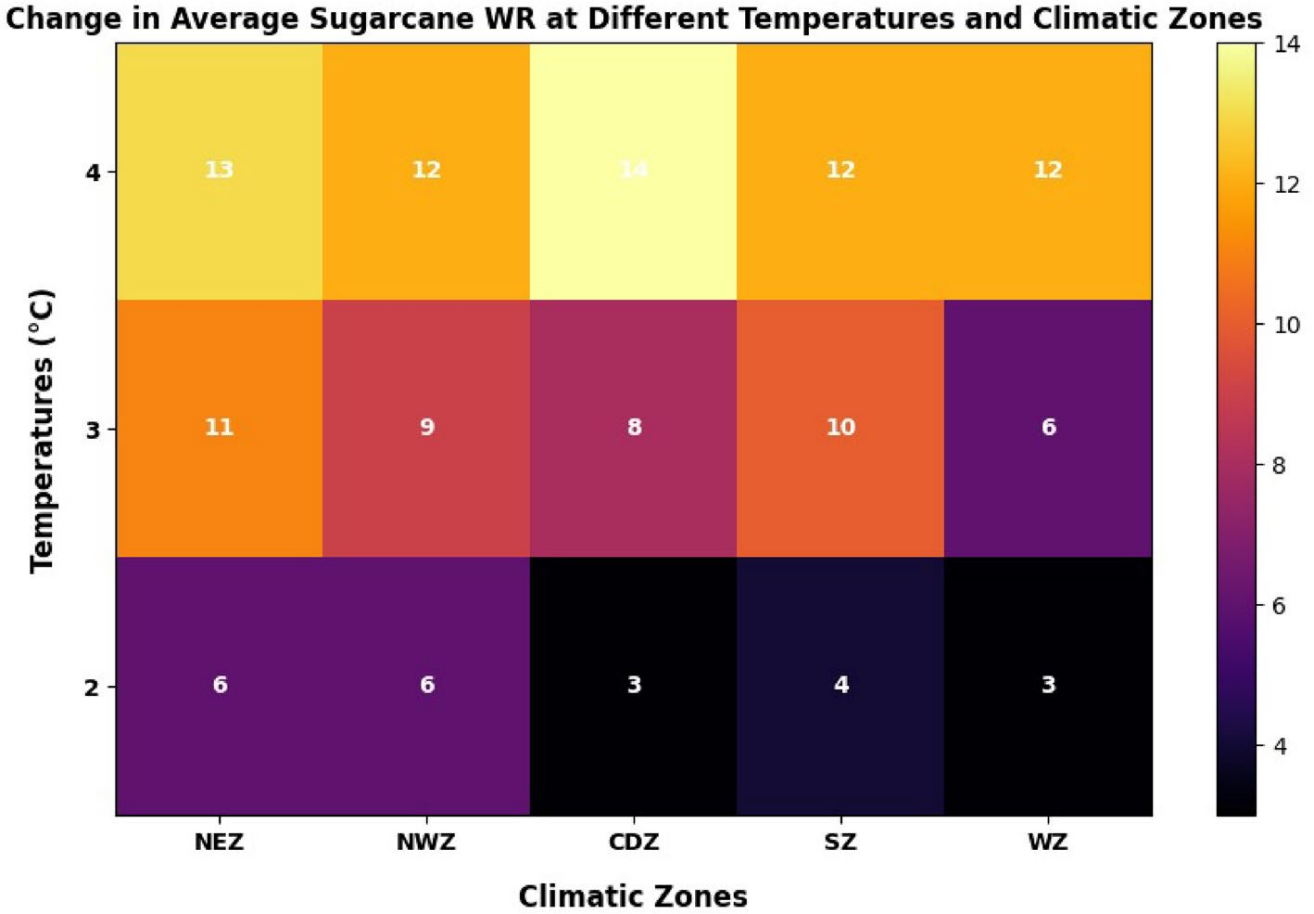
Elevated temperature effects on sugarcane water requirement.

The elevated temperature increased the WR in all the ACZ at varying magnitudes. An increase of 2 °C tends to lift the WR by an average of 4%. The elevated temperature by 3 °C resulted in a 9% increase in WR, and the 4 °C temperature rise increased the WR by 13% (Fig. 9).

As a result, there is an increased need for sugarcane in hotter climates. Understanding crop tolerance mechanisms and successfully implementing these technologies might greatly reduce the projected obstacles posed by climate change, while also meeting humanity's nutritional needs. There are two main sugarcane-producing regions, and the effects of future climate change and harsh weather on sugarcane yield differ significantly across them. Climate extremes in Southern China are not expected to alter in the upcoming decades. However, median yield in São Paulo state, Brazil, is projected to fall, and that the catastrophic effects of climate change would further reduce minimum yields. Sugarcane agriculture in Brazil hence carries a far higher risk of climate change^19^. In order to protect global sugarcane output from future climate risks, mitigation initiatives aimed at addressing local climate drivers are necessary. More research should concentrate on how sugarcane phenology responds to climate, especially the relationships between development rate, senescence of the leaves, and climate.

## Conclusion

Sugarcane yield varied across different agroclimatic zones of Tamil Nadu, with a maximum yield of 100.3 t/ha in the North eastern zone and a minimum of 94.2 t/ha in Southern zone. Higher WR (1700 mm) was noticed for the North eastern zone and less WR (1540 mm) for the southern zone compared to other agro-climatic zones of Tamil Nadu. Elevated temperatures substantially reduced the sugarcane yield, predicted to decrease by 3, 5, and 9% under 2 °C, 3 °C, and 4 °C elevated temperatures. Water requirement of sugarcane increased by 13% for the 4 °C rise in temperature. As a result of climate change, sugarcane yield is reduced, both vegetatively and reproductively.

### Supplementary Information

Supplementary Information 1.

Supplementary Information 2.

## Data Availability

The datasets generated and/or analysed during the current study are available in the IMD Gridded data repository, https://www.imdpune.gov.in/
